# Application of the Richards function to serum antibody titration

**DOI:** 10.3389/fimmu.2025.1657633

**Published:** 2025-12-12

**Authors:** Ágnes Kovács, Krisztián Papp, József Prechl, Tamás Pfeil

**Affiliations:** 1 Department of Biostatistics, University of Veterinary Medicine Budapest, Budapest, Hungary; 2 Department of Applied Analysis and Computational Mathematics, Eötvös Loránd University, Budapest, Hungary; 3 R&D Laboratory, Diagnosticum Zrt, Budapest, Hungary; 4 Department of Physics of Complex Systems, Eötvös Loránd University, Budapest, Hungary; 5 National Laboratory for Health Security, Institute of Mathematics, Eötvös Loránd University, Budapest, Hungary; 6 HUN-REN–ELTE Numerical Analysis and Large Networks Research Group, Budapest, Hungary

**Keywords:** logistic function, Richards function, growth, serum antibody, binding, antigen, affinity, thermodynamic titer

## Abstract

Conventional approaches to the titration of serum antibody binding use mid-point or end-point titers that are in a relative space and are therefore difficult to standardize. Here we propose the use of the thermodynamic titer, which, under appropriate measurement conditions, is a universal measure of the thermodynamic activity of serum antibodies. We show that the interpretation of the generalized logistic function as applied to biochemical binding events is possible using analogies to relative and absolute growth rates and size, which applies to the products of the reaction. Such deeper interpretation reveals the biological meaning of the asymmetry parameter of the function as a proportionality factor to ideal binding conditions. The use of a universally applicable and thermodynamically meaningful serum antibody titer could improve systematic mapping and understanding of antibody function.

## 1 Introduction

Antibody titration is similar to chemical titration in the sense that serially diluted serum is added to a reaction containing antigen. Unlike a chemical titration, the concentration of antibodies in serum is unknown and the concentration of antigen in the reaction is not necessarily known. As a consequence, the only way of expressing the result is a unitless index, the titer, which is expressed as the reciprocal of the dilution factor. The value of the titer for a serum antibody that reaches the predefined attributes when diluted 1:5000 is thus 5000. There are several approaches to characterize the titer (1). Endpoint titration considers the highest dilution where there is a positive signal (2). Midpoint (also called halfway) titration identifies the dilution factor where the signal falls to 50% of its starting value, often by fitting functions (3). By fitting a function to the binding curve one can interpolate to identify the dilution factor with greater precision. The use of antibody standards with known concentration allows transformation of titers into biochemical units of concentration. However, since monoclonal antibodies, their mixes or polyclonal antibody mixtures cannot precisely mimic a serum antibody binding curve these approaches are not accurate. The reason for this is the heterogeneity of an immune response: many different B-cell clones respond to an antigenic challenge and the composition of antibodies with respect to structure, affinity and concentration of these clones can be extremely variable.

The titration curve is a sigmoid growth curve that increases at an increasing rate until its inflection point and then increases at a decreasing rate approaching its upper limit. One of the first curves used to describe limited growth is the logistic curve, which is the graph of the logistic function first introduced by Verhulst (4). The various parametrizations of the family of logistic functions are known to immunologists as the four-parameter logistic function or 4PL (5, 6), as the Hill-equation for biochemists fitting ligand binding assays (7–9), and as the Langmuir-equation for modeling surface adsorption for biophysicists (10). From the theoretical point of view logistic functions model calibrated binary classification problems (11). Binary here refers to either being bound or unbound (free) and calibration means we assess the probability of these two states using a calibrated chemical force, the concentration of one of the reactants.

Because of the above discussed issues with standardization, most of the serological assays for specific antibodies express results in arbitrary units, which are not comparable across platforms, antigens and not even antibody isotypes. To circumvent the problem of obtaining results in incomparable arbitrary units we designed a method where antigen with known concentration is titrated simultaneously with serum antibody titration. This dual-titration approach built on the unique stoichiometry of microspot reactions and the practicality of running several reactions on the small surface area of a protein microarray (12). To fit the binding curves in two dimensions and to introduce flexibility into the binding curves of dual titration we applied the combination of two generalized logistic functions (13). Here we describe the rationale of our mathematical approach, show the analogies with growth curves and propose a general use for the Richards function in serum antibody titration.

## 2 Sigmoid functions and functions derived from sigmoid functions

### 2.1 Sigmoid functions

Sigmoid functions are increasing functions that have horizontal asymptotes at both ends and a single inflection point where the function is convex to the left and concave to the right. In applications, the left asymptote is usually zero. Many authors have summarized the sigmoid functions used in biological models and their properties (14–18, 25). Sigmoid functions are used, among others, to describe the size of finitely growing populations as a function of time (4) and the change in body sizes (19) or tumor sizes (20) also as a function of time. In immunochemistry, the concentration of the immune complex formed in antibody-antigen reactions is a sigmoid function as a function of both the logarithm of the antibody concentration and the logarithm of the antigen concentration (12, 21), in contrast to the exponential dependence obtained in the simple polyclonal model assuming constant antibody concentration (33) as a function of the logarithm of antibody concentration.

The definition of the sigmoid function only gives conditions for the shape of the graph of the function. We obtain more well-founded functions as solutions to the differential equation of the mathematical model that describes the phenomenon, because there is not only shape similarity between the experimental data curve and the graph of the function, but the graph also represents a solution to the differential equation that describes the dynamics of the phenomenon.

The most commonly used sigmoid functions in biology are the logistic function (22, 23), the Gompertz function (20, 24), and the Richards function (19). A special case of the Richards function is the logistic function, and its limiting case is the Gompertz function, and all of these are derived from differential equation models (13, 14, 16–18, 25). We mention that many continuous probability distribution functions are sigmoid, e.g. the normal and, for certain parameters, the Weibull.

The logistic function is

(1) $$L\left(x\right)=\frac{A}{1+be^{-kx}}$$

where A,b,k>0, and its graph, the logistic curve, is symmetric about its inflection point. Here *A* is the limit at infinity or upper limit, *k*>0 is the approximate exponential growth rate at minus infinity, and *b* is the shift parameter. If we set $b=e^{kx_{c}}$ then


$$L\left(x\right)=\frac{A}{1+e^{-k\left(x-x_{c}\right)}}$$

and with this parametrization the real number *x_c_* is the inflection point of the function. This family of functions is the general solution of the logistic differential equation.


$$\frac{dL}{dx}=kL\left(1-\frac{L}{A}\right)$$

with a range of values between 0 and *A*. If an additive constant is included, we get the four-parameter logistic function or 4PL as a function of e^*x*^ which allows for a non-zero left asymptote (see the **Supplementary Materials**).

We get a natural generalization of the logistic function (Equation 1) by raising its denominator to a power, which is called the Richards function or generalized logistic function.


$$R\left(x\right)=\frac{A}{{\left(1+be^{-kx}\right)}^{m}}$$

**Figure 1:**
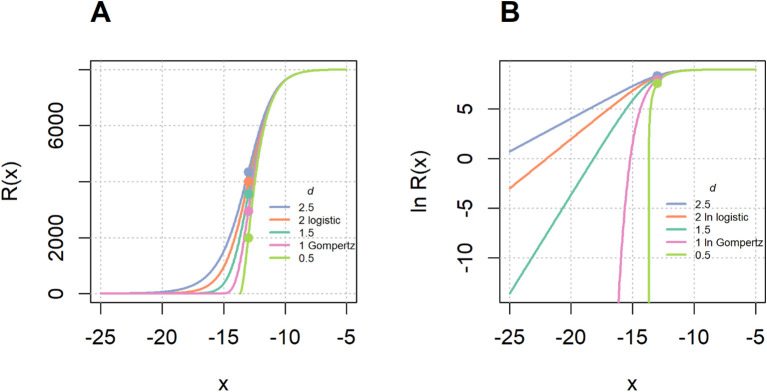
Richards growth function on the lin–lin scale **(A)** and the log–lin scale **(B)** for different shape parameters d, with all other parameters held constant. The x-axis represents the independent variable x (e.g., logarithm antigen concentration or logarithm serum dilution). The dots denote the inflection points for each curve.


For this function, A>0 is the upper limit, and for k,m>0, km is the rate of the initial approximately exponential growth. Unfortunately, the function given in the form


$$R\left(x\right)=\frac{A}{{\left(1+e^{-k\left(x-x_{c}\right)}\right)}^{m}}$$

has an inflection point at *x*_c_ only if m=1. Therefore, a commonly used form of the Richards function for m>0 is

(2) $$R\left(x\right)=A{\left(1+\left(d-1\right)e^{-k\left(x-x_{i}\right)}\right)}^{\frac{1}{1-d}}$$

where *A*>0 is the upper limit, $\frac{k}{d-1}>0$ is the rate of the approximate exponential growth at minus infinity, *d*>1 is the asymmetry parameter and the real number *x_i_* is the inflection point of the function (**Figure 1**). The ratio of the value at the inflection point and the upper limit (in short inflection rate value)

$$\frac{R\left(x_{i}\right)}{A}=d^{\frac{1}{1-d}}$$

depends only on the parameter *d*, and varies between $\frac{1}{e}$ (limit value as d→1, the limit function is the Gompertz function) and 1 (limit value as d→∞), and *d* = 2 returns the logistic function.

For 0 < *d* < 1, the Richards function (Equation 2) is usually defined starting from its zero point, in an interval infinite from the right. Its inflection rate value changes between 0 and $\frac{1}{e}$.

If we add an arbitrary constant to the Richards function, we obtain the five-parameter logistic function 5PL as a function of e^*x*^ (see the **Supplementary Materials**).

In our work, we use the parameter ν=d−1instead of *d* of the Richards function when ν > 0 as in (13, 15, 25, 26):

(3) $$R\left(x\right)=A{\left(1+\nu e^{-k\left(x-x_{i}\right)}\right)}^{-\frac{1}{\nu }}$$

Here *A* > 0 is the upper limit, $\frac{k}{\nu }>0$ is the rate of the approximate exponential growth at minus infinity, and ν is a shape parameter determining the inflection rate value ${\left(\nu +1\right)}^{-\frac{1}{\nu }}$.

These functions are solutions of the differential equation.


$$\frac{dR}{dx}=\frac{k}{\nu }R\left(1-{\left(\frac{R}{A}\right)}^{\nu }\right)$$

The slope, or rate of growth, of a sigmoid function is greatest at its inflection point. Since this point is an important location also for the Richards function, it is reasonable to normalize the function at the inflection point, i.e., to achieve a value of 1 at the inflection point using the appropriate constant multiplier $\frac{{\left(1+\nu \right)}^{\frac{1}{\nu }}}{A}$ (13, 26):


$$R_{\nu }\left(x\right)={\left(\frac{1+\nu }{1+\nu e^{-k\left(x-x_{i}\right)}}\right)}^{\frac{1}{\nu }}$$

For various parametrizations and other properties of the sigmoid functions discussed above, see the **Supplementary Materials**.

### 2.2 Functions derived from sigmoid functions

In our work, we take the logarithmic version of the Richards function, Equation 3, which yields

(4) $$\text{ln}R\left(x\right)=\text{ln}A-\frac{1}{\nu }\text{ln}\left(1+\nu e^{-k\left(x-x_{i}\right)}\right)$$

when evaluating the measurement results. This is a concave function with an upper asymptote of ln*A* and a leftmost asymptote with a slope of $\frac{k}{\nu }$.

A double Richards function is a two-variable function that is the product of a Richards function of each of the two variables. A common rate parameter k is used for both terms to represent identical slope behavior, consistent with the symmetry assumed in our experimental framework.

(5) $$R\left(x,y\right)=R_{1}\left(x\right)R_{2}\left(y\right)=C{\left(1+\nu_{1}e^{-k\left(x-x_{i}\right)}\right)}^{-\;\frac{1}{\nu_{1}}}{\left(1+\nu_{2}e^{-k\left(y-y_{i}\right)}\right)}^{-\;\frac{1}{\nu_{2}}}$$

Its natural logarithm is


$$\ln R\left(x,y\right)=\text{ln}\left(R_{1}\left(x\right)R_{2}\left(y\right)\right)=\text{ln}C-\frac{1}{\nu_{1}}\text{ln}\left(1+\nu_{1}e^{-k\left(x-x_{i}\right)}\right)-\frac{1}{\nu_{2}}\text{ln}\left(1+\nu_{2}e^{-k\left(y-y_{i}\right)}\right)$$

Both terms can be normalized to their own inflection points:

(6) $$R_{n}\left(x,y\right)=R_{1}\left(x\right)R_{2}\left(y\right)=C_{n}{\left(\frac{1+\nu_{1}}{1+\nu_{1}e^{-k\left(x-x_{i}\right)}}\right)}^{\frac{1}{\nu_{1}}}{\left(\frac{1+\nu_{2}}{1+\nu_{2}e^{-k\left(y-y_{i}\right)}}\right)}^{\frac{1}{\nu_{2}}}$$

and

(7) $$\ln R_{n}\left(x,y\right)=\text{ln}\left(R_{1}\left(x\right)R_{2}\left(y\right)\right)=\text{ln}C_{n}+\frac{1}{\nu_{1}}\text{ln}\left(\frac{1+\nu_{1}}{1+\nu_{1}e^{-k\left(x-x_{i}\right)}}\right)+\frac{1}{\nu_{2}}\text{ln}\left(\frac{1+\nu_{2}}{1+\nu_{2}e^{-k\left(y-y_{i}\right)}}\right)$$

with the visual advantage of moving $x_{i}$ and $y_{i}$ to the same horizontal line.

## 3 Graphical presentation of Richards curves

**Figure 2:**
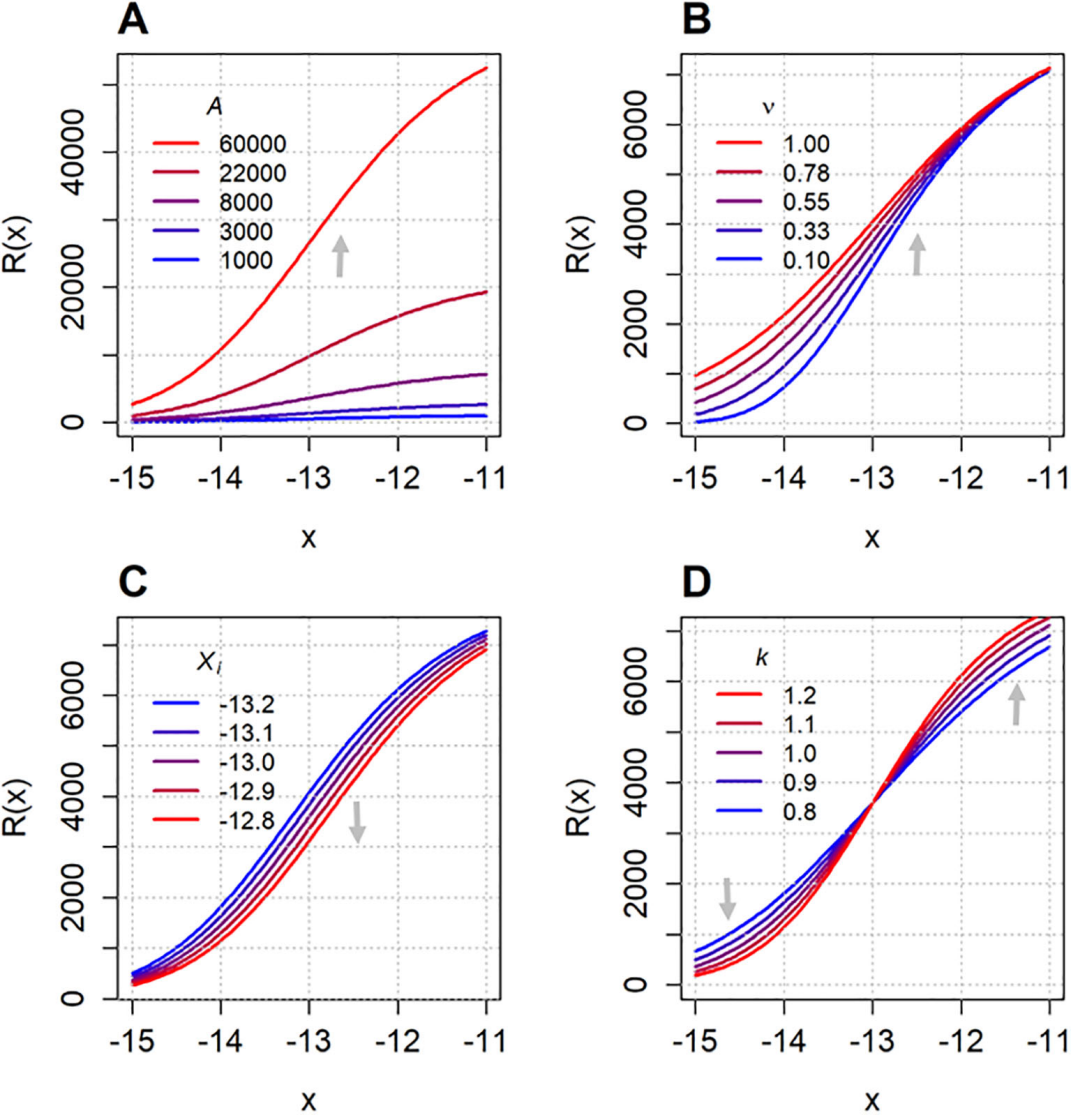
The Richards function (Equation 3) plotted as each of its parameters – A**(A)**, ν**(B)**, $x_{i}$**(C)** and k**(D)** – is varied individually. The fixed parameter values are A=8000, ν=0.5, $x_{i}=-13$ and k=1, while the varying parameter values are specified in each corresponding subplot. The x-axis range (–15 to –11) corresponds to the range of log transformed concentrations used in the experimental dataset introduced later in Section 3.

**Figure 2** shows how the Richards function (Equation 3) behaves as its parameters ( A, ν, $x_{i}$ and k) are varied individually. Increasing A proportionally shifts the function vertically, raising its upper asymptote. The asymmetry parameter ν controls the steepness of the curve: higher values of ν give less steep transitions between the asymptotes. Varying the inflection point $x_{i}$ results in a horizontal shift of the curve, i.e., increasing $x_{i}$ moves the entire sigmoid curve to the right. When A and ν remain the same, then regardless of the value of k, $R\left(x_{i}\right)$ will be the same. A larger k implies a more rapid change, it determines how gradual the transition of the sigmoid curve is. Similar presentation is seen in (5).

**Figure 3:**
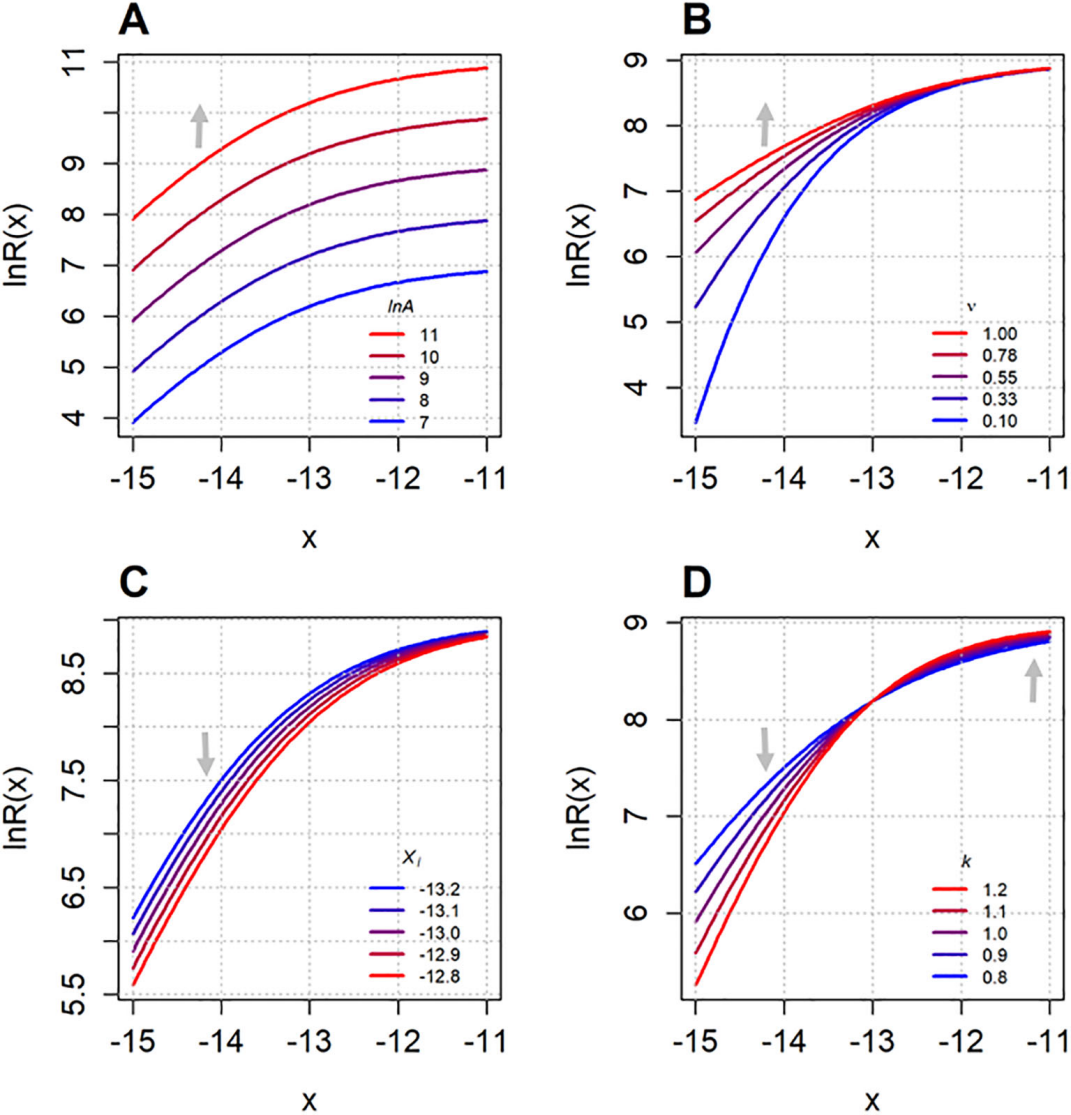
The Richards function (Equation 3) plotted on the logarithmic scale (Equation 4) as each of its parameters – lnA**(A)**, ν**(B)**, $x_{i}$**(C)** and k**(D)** – varied individually. The fixed parameter values are lnA=9, ν=0.5, $lnx_{i}=-13$ and k=1. The varying parameter values are specified in each corresponding subplot. The x-axis range (–15 to –11) corresponds to the range of log transformed concentrations used in the experimental dataset introduced later in Section 3.

Similarly **Figure 3** shows how the Richards function (Equation 3) behaves on the logarithmic scale (Equation 4) as its parameters ( lnA, ν, $x_{i}$ and k) are varied individually. Increasing lnA shifts the function vertically, raising its upper asymptote. The asymmetry parameter ν controls the steepness of the curve: higher values of ν produce curves that approach the asymptotes more slowly on one side. Varying the inflection point $x_{i}$ results in a horizontal shift of the curve, while changes in k determines how gradual the transition of the sigmoid curve is.

### 3.1 Illustration using experimental data

To illustrate the Richards model applicability we fitted it to experimental data obtained from a representative serum sample PS332, which was part of the commercially available set of serum samples from individuals with PCR confirmed SARS-CoV-2 infection from Raybiotech (Peach Tree corners, Georgia, USA). IgG reactivity against the RBD domain of the virus was measured as described previously (27). The reactivity of IgG against the tested viral antigen was confirmed using the experimental technology that builds on our model and simultaneously fits two Richards functions to the measurement data. The example is intended for methodological demonstration rather than biological generalization.

**Figure 4:**
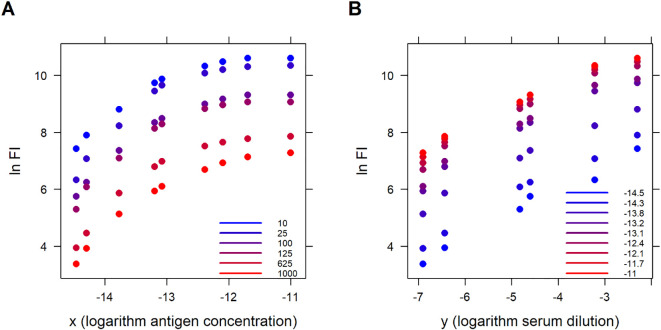
Experimental data (IgG 332) is presented in two dimensions. Logarithm of fluorescence intensity ( lnFI) vs logarithm antigen concentration ( x) colored with different serum dilutions **(A)** and logarithm of fluorescence intensity ( lnFI) vs logarithm serum dilution ( y) colored with different antigen concentrations **(B)**.

Data are best visualized by plotting the logarithm of fluorescence intensity ( lnFI) vs logarithm antigen concentration ( x) colored with the different serum dilutions, and the logarithm fluorescence intensity ( lnFI) vs serum dilution ( y) colored with the different antigen concentrations (**Figure 4**)

**Figure 5:**
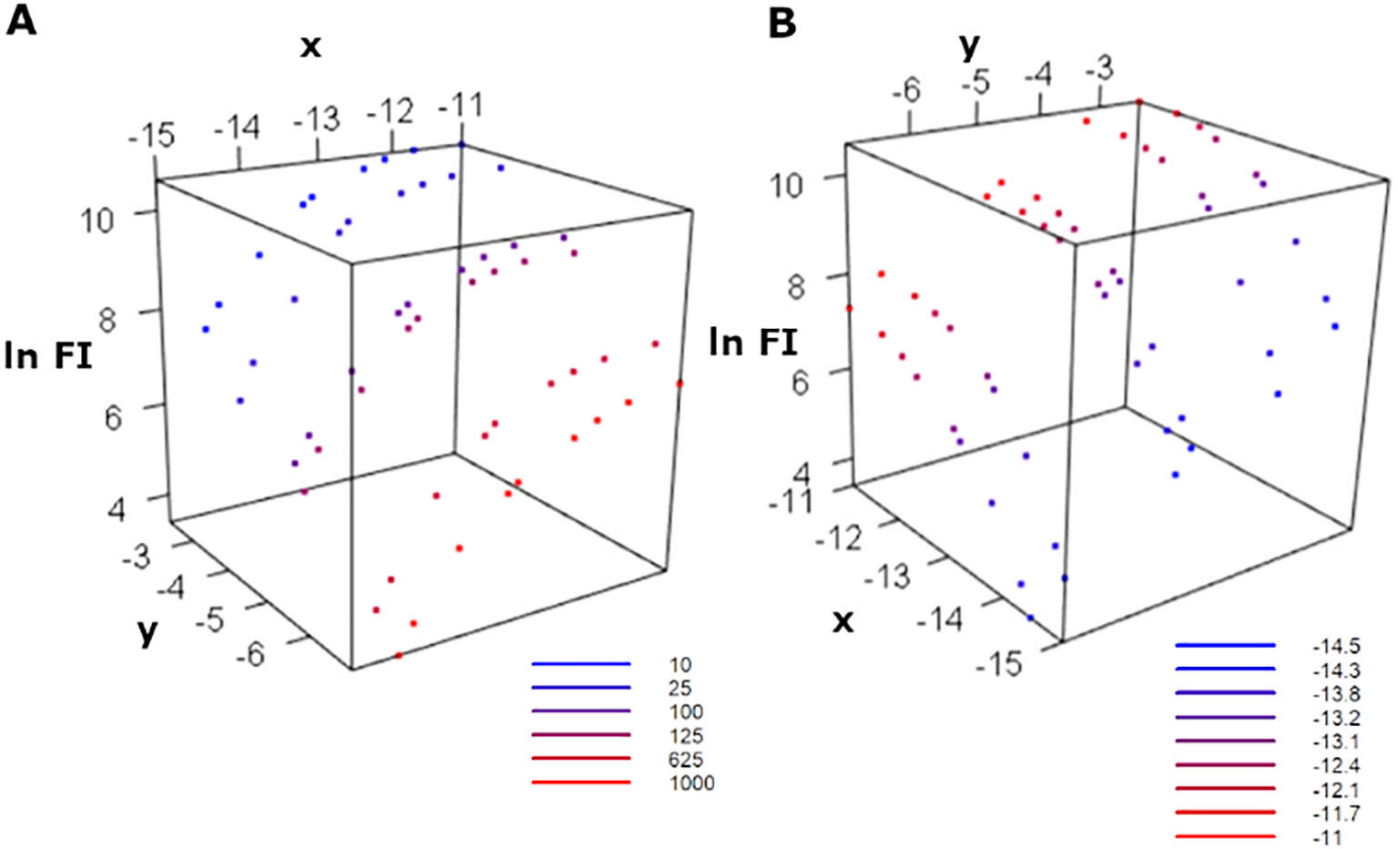
Experimental data (IgG 332) is presented in three dimensions. Measurements of logarithm of fluorescence intensity ( lnFI) vs logarithm antigen concentration and logarithm serum dilution colored with different serum dilutions **(A)** and colored with different antigen concentrations **(B)**.

In addition, these data can also be presented in 3 dimensions as shown in **Figure 5**.

### 3.2 Statistical performance

The estimation of the Richards parameters is feasible with standard nonlinear least squares methods, and it generally provides robust fits especially when the full S-shaped curve is well sampled. However, convergence issues can arise when data are sparse or when the inflection point is poorly sampled, which can affect the stability and reliability of parameter estimates. Since the literature on the statistical properties of the single Richards curve is already extensive, we now focus on the statistical performance of the double Richards model.

### 3.3 Statistical performance of the double Richards model

Unlike simpler sigmoidal models, the double Richards model (Equation 5) requires the estimation of two sets of nonlinear parameters, which presents both computational and interpretive challenges. We applied nonlinear regression to fit the double Richards model and additionally, we incorporated Bayesian inference to improve the estimating procedure.

Fitting the double Richards model relies on iterative nonlinear optimization algorithms, typically the Levenberg-Marquardt method, which poses significant statistical challenges. Achieving convergence in nonlinear regression is not guaranteed, particularly for complex models like the double Richards, where parameter spaces are large and often have flat or multimodal likelihood surfaces.

In our experience, naive initialization of parameters frequently led to convergence failure or biologically implausible estimates. To improve convergence reliability, we implemented a data-driven procedure to generate starting values tailored to the structure of the double Richards function. Central parameters were set using medians or relevant quantiles, depending on the skewness of the variables, while shape parameters were set to 1. This approach provided reasonable starting values across our datasets and helped mitigate common convergence failures.

We also considered other techniques such as using derivatives and piecewise regression but even with these procedures convergence was not universal, especially in datasets with high noise levels or limited range. However, we also note that having good initial parameter estimates does not guarantee convergence in nonlinear regression. Even when starting values are close to the true parameters, convergence may fail due to the complex (often flat) shape of the likelihood surface.

Despite obtaining good starting values, convergence was not guaranteed due to the model’s complexity. Therefore, we also employed a Bayesian estimation framework using weakly informative priors. Parameter inference was performed via Markov Chain Monte Carlo (MCMC) sampling, which provided greater stability and more reliable estimates, especially when the likelihood surface was complex or multi-modal.

### 3.4 Illustration of the double Richards model using experimental data

To demonstrate the applicability and performance of the double Richards model, we fitted it to the experimental biding data (serum sample PS332 from an individual with PCR confirmed SARS-CoV-2 infection, having measured the IgG reactivity against the RBD domain of the virus) introduced in Section 3.1. Parameter estimates were obtained using both nonlinear least squares and Bayesian methods as shown in **Table 1**.

**Table 1:** Parameter estimation from the double Richards model (Equation 7) are given using both nonlinear least squares and Bayesian methods using the IgG PS332 data.

Method	lnC	$\nu_{1}$	$lnx_{i}$	$\nu_{2}$	$lny_{i}$
Nonlinear least squares	9.75	0.08	-12.92	0.99	-2.97
Bayes estimation	9.77	0.09	-12.93	1.00	-2.93

To demonstrate the performance of the double Richards model in fitting complex sigmoidal curves, we applied it to experimental binding data obtained from a serum sample (PS332). This sample was derived from an individual with PCR-confirmed SARS-CoV-2 infection, and the IgG antibody reactivity was measured against the receptor-binding domain (RBD) of the virus. The data, introduced in Section 3.1, displays a biphasic response characteristic, making it a suitable candidate for modeling with a double-sigmoid function.

**Figure 6:**
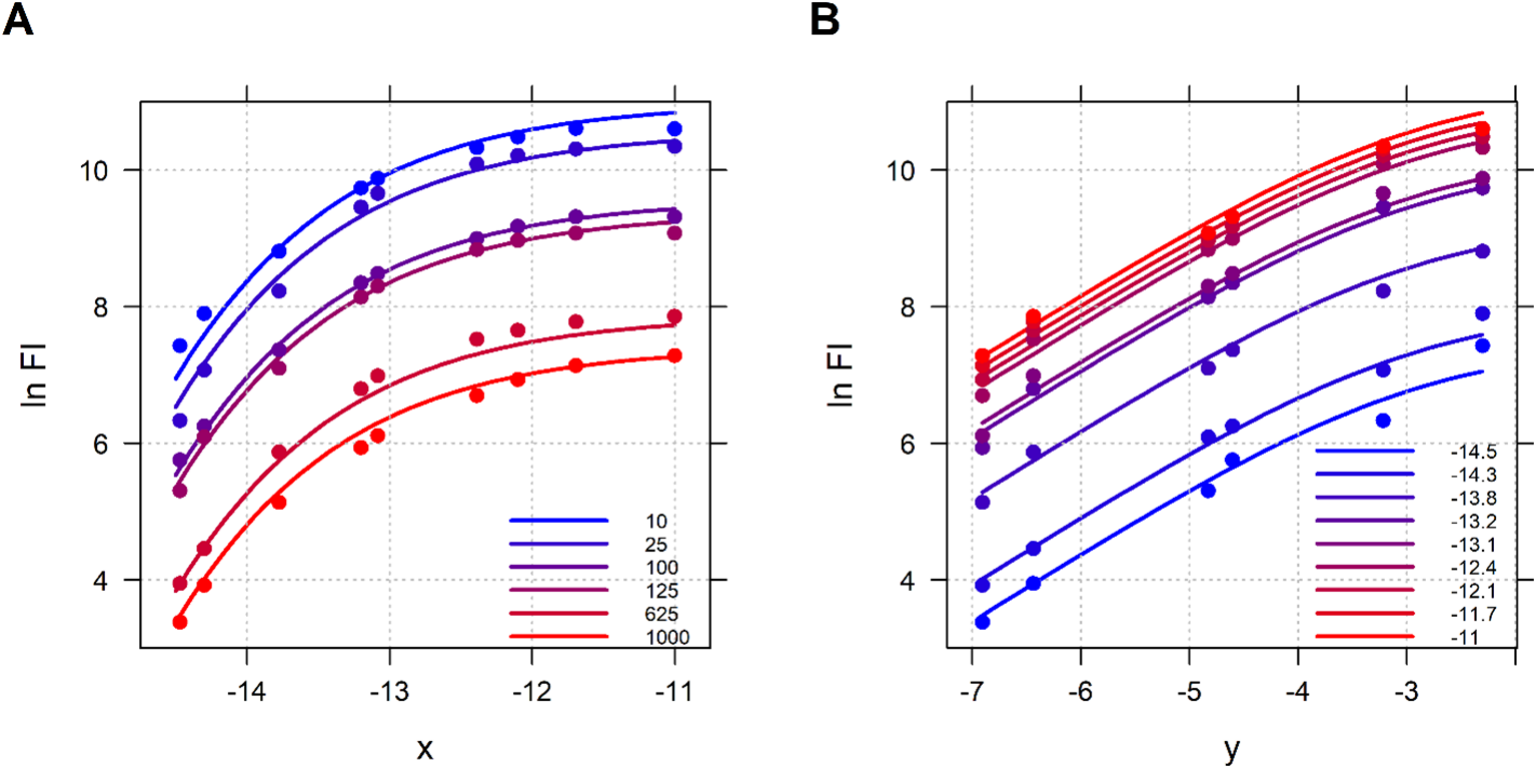
The double Richards function (Equation 6) is plotted on the logarithm scale (Equation 7) with parameters listed in **Table 1**, shown together with the corresponding experimental data (IgG 332). Logarithm of fluorescence intensity (ln FI) vs logarithm of antigen concentration (x) colored by serum dilution **(A)** and logarithm of fluorescence intensity (ln FI) vs logarithm of serum dilution (y) colored by antigen concentration **(B)**.

**Figure 6** shows the estimated fluorescence intensity based on the above model.

**Figure 7:**
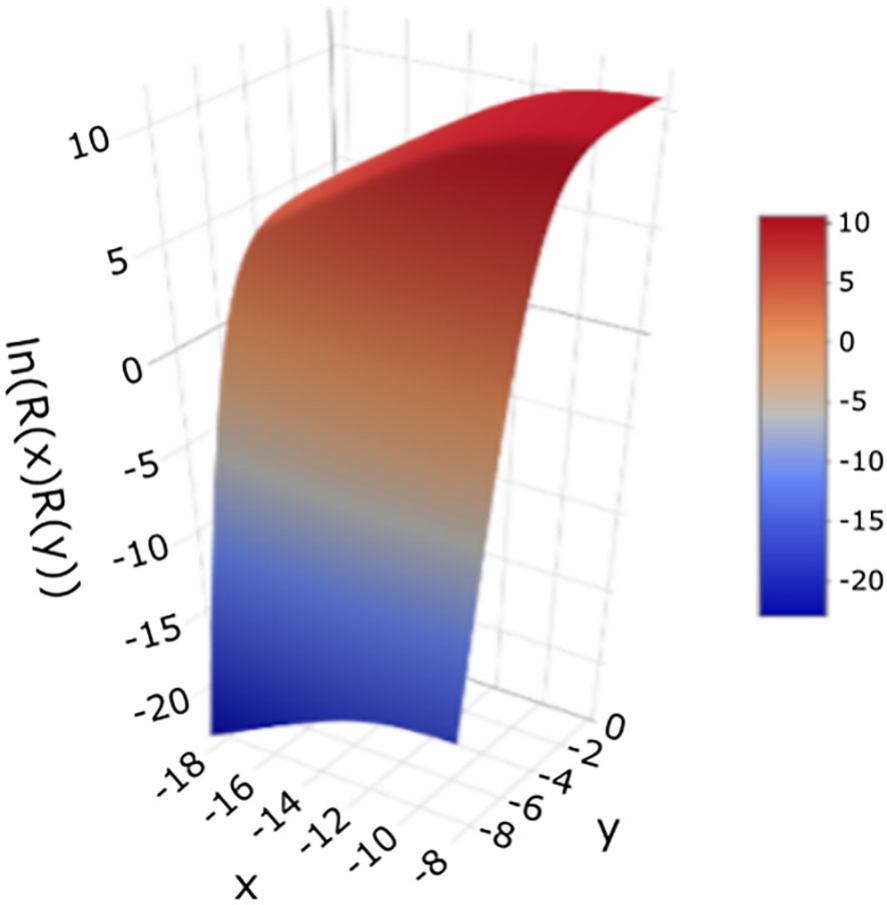
Three-dimensional plot of the double Richards function (Equation 6) on a logarithmic scale (Equation 7), showing the logarithm of fluorescence intensity as a function of the logarithm of antigen concentration and logarithm of serum dilution. Parameter values were estimated from the experimental data (**Table 1**).

To further illustrate the behavior of the fitted model, these data are also presented in 3-dimensions (**Figure 7**), showing how the modeled response (logarithm of fluorescence intensity) changes concurrently with both independent variables - the logarithm of antigen concentration and the logarithm of serum dilution. This representation highlights the interaction between the two variables and the overall shape of the fitted surface.

Experimental data (IgG 332) is presented in three dimensions. Measurements of logarithm of fluorescence intensity ( lnFI) vs logarithm antigen concentration and logarithm serum dilution colored with different serum dilutions (A) and colored with different antigen concentrations (B).

## 4 Application of logistic functions to biochemical reactions

### 4.1 Analogy to growth curves

Sigmoid growth curves describe cumulative processes: cells accumulate forming increasing mass, trees accumulate creating forests, individuals accumulate resulting in growth of populations (14, 15, 28, 29). Such biological growth is observed as a function of time. During biochemical reactions products of the reaction can also accumulate, their concentration grows, however, titration curves are expressed as a function of chemical forces instead of time. In our case, when the biochemical reaction is the binding of Ab and Ag molecules, these chemical forces are expressed as the logarithms of the concentration of the titrated component. The reason for taking the logarithm is to obtain quantities related to chemical energy or chemical potential. The observation of growth here therefore does not mean the observing of change of product concentrations of a given reaction in time but rather the equilibrium product concentrations from several distinct reactions where different chemical energies were applied. In each of these different measurements the reaction has to reach equilibrium in order to make them comparable and suitable for analysis. This “growth curve” is the binding curve, with its discrete measurement points in the dimension of chemical potential instead of time.

**Figure 8:**
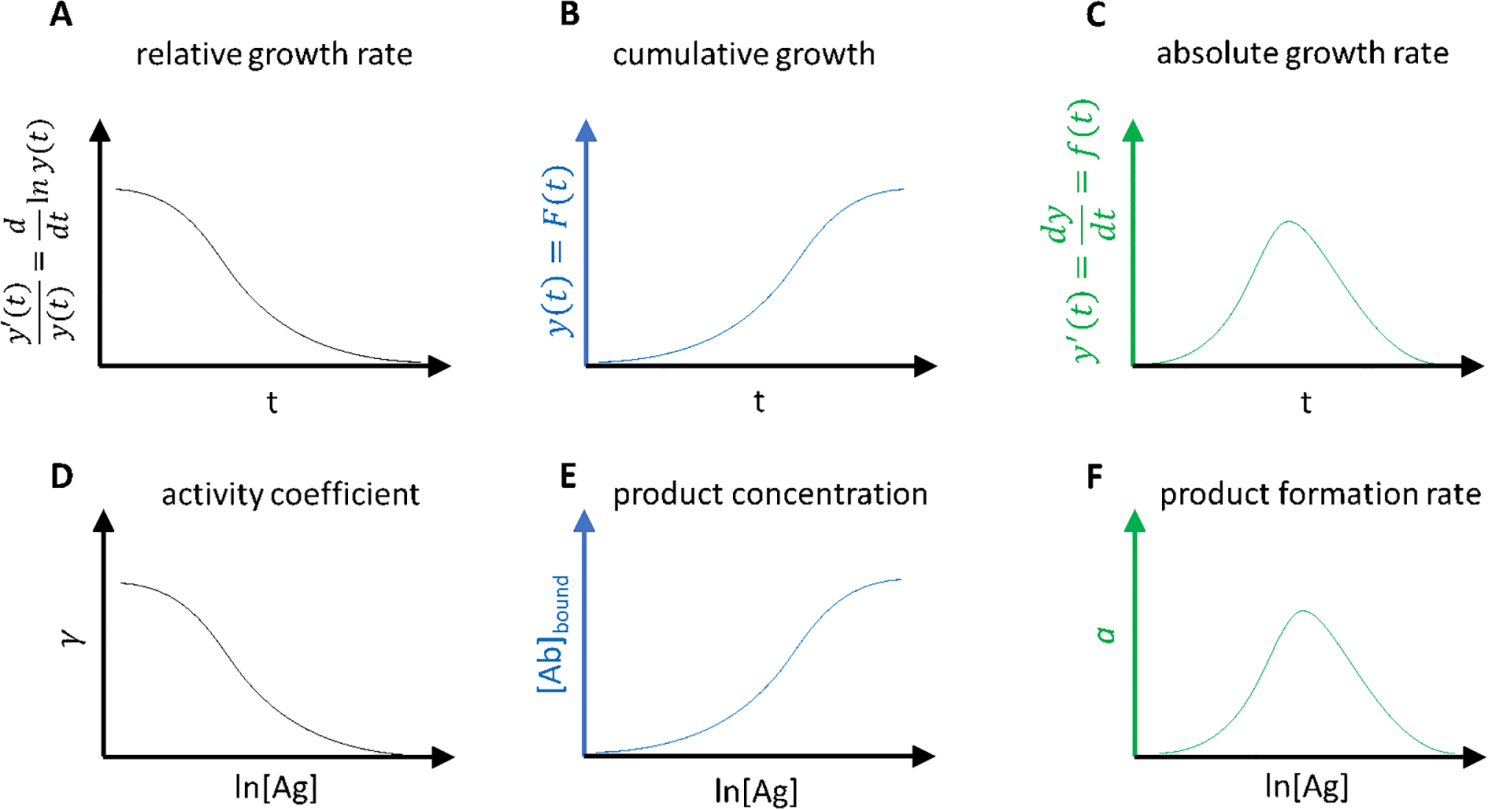
Relationship between curves of growth **(A-C)** and biochemical reaction **(D-F)**. Analogies are apparent between pairs **(A, D, B, E, C, F)** of related phenomena.

When modelling growth with logistic functions, two important and useful transformations of the growth function can be made (30). The derivative of the function, which describes absolute growth rate, tells us how fast the concentration of products is increasing as we increase the chemical force applied. Even more useful is the relative growth rate, which is described by the derivative of the logarithm of the growth function. Relative growth rate is the rate of change of size compared to current size. This is a monotonously decreasing function, as the size increases the relative rate of growth slows down. This is because the resources supporting the growing entity gradually become smaller and smaller in comparison to the needs of the entity. For a binding reaction that is interpreted as the waning of chemical energy supporting the reaction against the energies counteracting product formation. In other words, this function represents how the titrated chemical energy is used up for supporting the reaction (**Figure 8**). It is a mathematical identity that the product of the growth function and the relative growth rate function yields the absolute growth rate function. In thermodynamic terms that corresponds to the product of concentration ([Ab]bound) and activity coefficient (γ), yielding the relative thermodynamic activity (α).

### 4.2 Interpretation of deformation of the logistic curve

**Figure 9:**
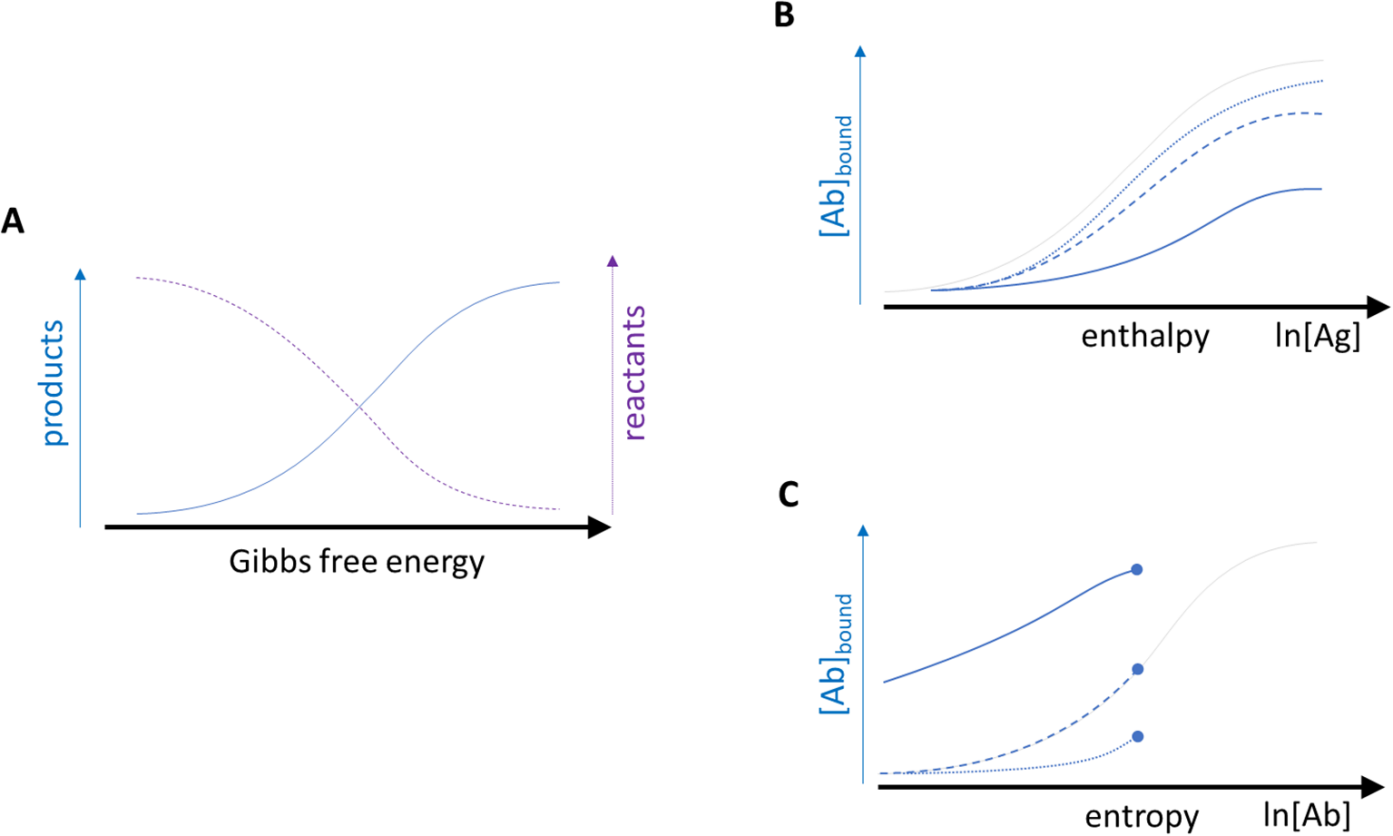
Deformation of ideal curves by observation bias. When product formation is a function of free energy **(A)**, then the titration of enthalpic **(B)** or entropic **(C)** components of binding reaction will result in deformations of the logistic curve. While Ag concentration can be varied over the relevant reasonable range **(B)**, serum titration starts with undiluted serum, represented by filled circles as end-points **(C)**.

Growth curves are not always as ideal as would be suggested by a logistic growth curve (**Figure 9A**). The generalization of the logistic function allows for deformations of the curve by the introduction of another parameter into the function: the asymmetry parameter. Non-ideality of binding curves has also been observed for immunoassays, where the fitting of the curves was also improved by adding another parameter to the function, transforming four-parameter logistic function (4PL) into five-parameter logistic function (5PL) (5, 31, 32). The introduction of the novel parameter yields functions that are solutions of the Richards differential equation, but the parametrization of these solutions has important consequences for the interpretation of biochemical meaning of the parameters (see (13, 33)).

From the molecular point of view the deformation of the binding curve means that the accumulation of products (proportional to the absorbed chemical energy) and the decrease in available energy are not symmetric: we are “losing” energy somewhere. This loss is only in the observation of course, and suggests that our model does not count with factors that contribute to the binding. Such an asymmetry in the measurement can arise when the measurement system itself is biased for the detection of certain forms of energy. Microspot immunoassays are also known as mass independent immunoassays because under appropriate conditions the total number of molecules (mass) is not affecting the measurement, only the concentration (thermodynamic activity). The advantages of mass independence can be exploited for antibody sandwich type measurements of antigen analytes (34, 35) and also for antigen specific serum antibody measurements (12). In a stricter thermodynamic sense microspot assays can allow the separation of the measurement of two thermodynamic potentials: enthalpy and entropy (**Figures 9B, C**) (36, 37). By varying the density of surface immobilized antigen molecules, we can adjust the number of binding sites for non-covalent bonding per unit area. That way we control the enthalpic forces contributing to binding. On the other hand, by varying the concentration of antibody molecules in solution, we adjust the number of ‘particles’ contributing to the filling of energy levels of the system. By particles here we mean antibody molecules, which populate the binding energy landscape, and which attain different energy levels by conformational changes. Thus, when titrating surface antigen density or antibody concentration in solution, we decouple the measurement of contribution of enthalpic and entropic forces to the binding.

### 4.3 Two Richards functions for two thermodynamic potentials

**Figure 10:**
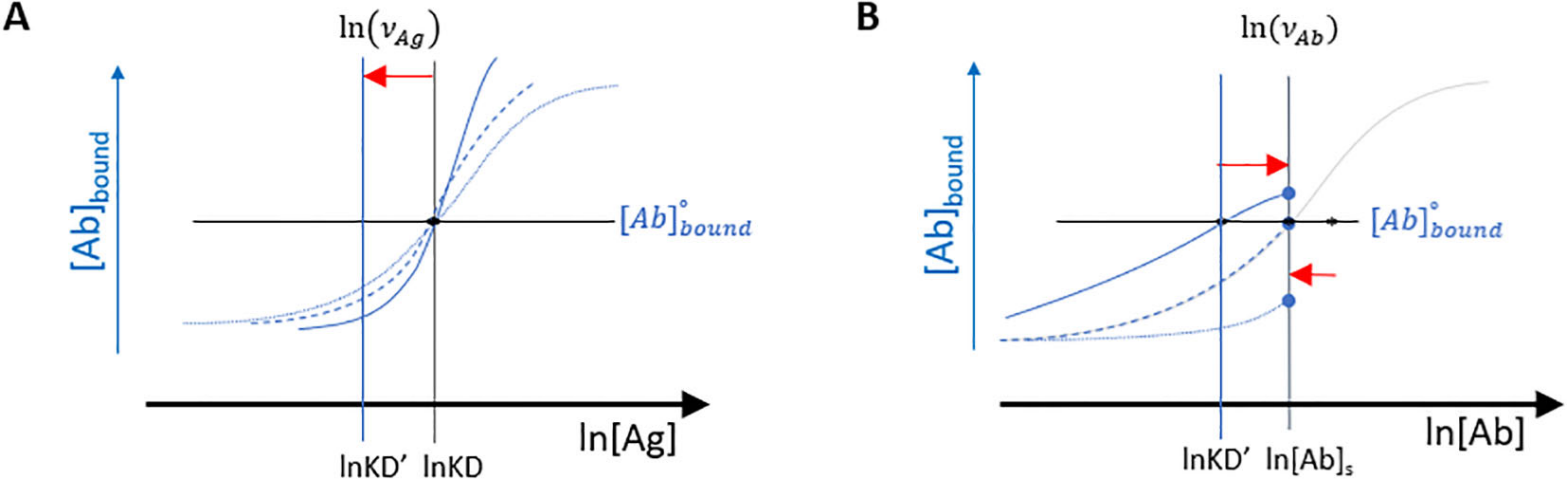
Effects of asymmetry parameters on normalized functions. The growth of bound Ab concentration, as a function of reaction enthalpy **(A)** and entropy **(B)**. Apparent affinity under these conditions is always greater than true affinity, (KD’< KD), therefore ln( $\nu_{Ag}$) is always negative. Excess entropy can both hinder or enhance binding, ln( $\nu_{Ab}$) can therefore be both positive or negative, corresponding to the observed total serum [Ab] being higher or lower than KD.

By decoupling the measurement of enthalpic and entropic contributions to binding, we can estimate the partial contribution of these thermodynamic potentials. Because these two chemical forces jointly contribute to binding, the estimation of the partial contribution of one results in solving the partial contribution of the other, as well. We can incorporate the deformation caused by enthalpy or entropy-biased measurements (**Figure 10**) into the asymmetry parameter of the Richards function as defined by Equations 8, 9.

(8) $$\text{ln}\left(\nu_{Ag}\right)=\text{ln}\left(\frac{KD'}{KD}\right)=\left(\Delta H-\Delta H{}^{\circ}\right)/RT=\Delta \Delta H/RT$$

(9) $$\text{ln}\left(\nu_{Ab}\right)=\text{ln}\left(\frac{{\left[Ab\right]}_{s}}{KD}\right)=\left(\Delta S-\Delta S{}^{\circ}\right)/R=\Delta \Delta S/R$$

where KD’ and KD are apparent and true equilibrium dissociation constants, [Ab]_s_ is the serum antibody concentration, ΔH° and ΔS° stand for ideal, equal contribution of enthalpy and entropy to binding, ΔH and ΔS are total enthalpic and entropic contributions to binding, while ΔΔH and ΔΔS are enthalpic and entropic parts of excess Gibbs free energy, respectively (38). Energies are expressed with reference to the universal gas constant R and thermodynamic temperature T.

Whilst the two asymmetry parameters could also be estimated separately, technically we can simultaneously measure and estimate the two contributions. This is carried out by creating a measurement matrix, where rows and columns correspond to binding signals obtained by titrating enthalpic and entropic forces, namely ln[Ag] and ln[Ab]. It is due to the measurement conditions that the titration of Ag and Ab reflect distinct chemical forces: Ag molecules are immobilized on a surface in the presence of huge excess of Ab in solution (12, 27). We can then fit two Richards curves with asymmetry parameters, defined by Equations 10, 11, simultaneously, one for Ag and one for Ab titration.

(10) $$R_{1,\nu }\left([Ag]\right)\sim {\left(\frac{1+\nu_{Ag}}{1+\nu_{Ag}e^{-k\left(\text{ln}\left[Ag\right]-\text{ln}\left[Ag\right]{}^{\circ}\right)}}\right)}^{\frac{1}{\nu_{Ag}}}$$

(11) $$R_{2,\nu }\left([Ab]\right)\sim {\left(\frac{1+\nu_{Ab}}{1+\nu_{Ab}e^{-k^{\prime }\left(\text{ln}\left[Ab\right]-\text{ln}\left[Ab\right]{}^{\circ}\right)}}\right)}^{\frac{1}{\nu_{Ab}}}$$

The fluorescent signal intensity obtained by measuring bound [Ab], $FI_{{\left[Ab\right]}_{bound}}$, is then given by the Equation 12

(12) $$FI_{{\left[Ab\right]}_{bound}}=FI_{Ab}*{\left[Ab\right]}_{bound}^{{}^{\circ}}*R_{1,\nu }\left([Ag]\right)*R_{2,\nu }\left([Ab]\right)$$

where $FI_{Ab}$ is the factor for converting bound [Ab] to fluorescence signals and can be obtained by calibration measurements.

## 5 Practical guide for using Richards curves for titration

Most of the immune assays in use today employ only a single Ag concentration. Therefore, if the condition of massive excess of Ab in solution is met by the assay, it is still possible to fit an Ab titration curve without an Ag titration curve. While the affinity cannot be addressed without Ag titration it is possible to obtain universally comparable values characterizing serum antibodies: the dilution factor of the undiluted serum from the point of inflection, given by $\nu_{Ab}$ is the thermodynamic titer, also called thermodynamic concentration, [Ab]s/K_D_. The thermodynamic titer is an indicator of the ability of antibodies to saturate Ag. When its value is 1, Ag is half saturated. Above one saturation increases, below one it decreases below 50%. This value is therefore independent of the affinity of serum antibodies.

**Figure 11:**
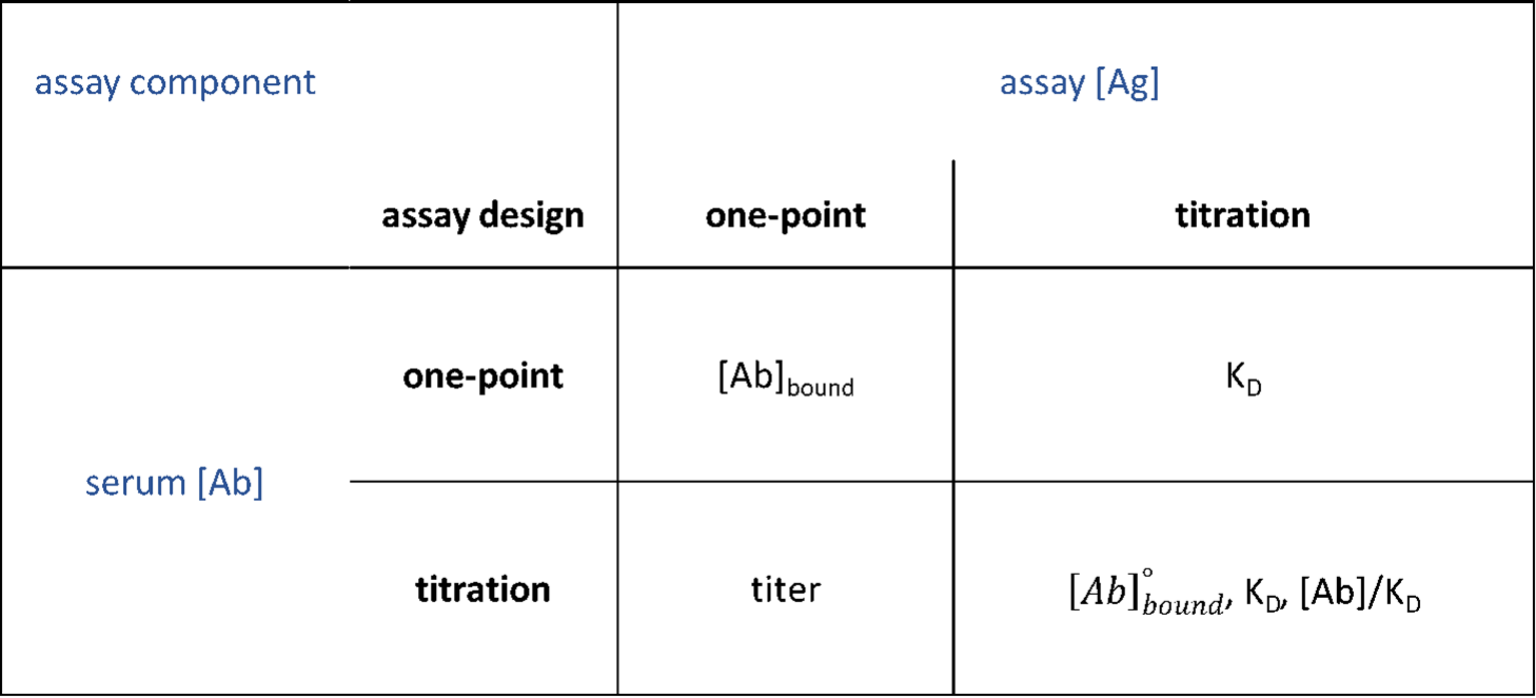
Comparison of one-point, titration and dual-titration assay outputs. Standard binding site occupancy ${\left[Ab\right]}_{bound}^{{}^{\circ}}$, K_D_, equilibrium dissociation constant.

By calibrating the signals representing Ab binding we can convert signal intensity to bound Ab concentration, [Ab]_b_. This approach is used in one-point assays for obtaining results with concentration units, but it should be noted that the proper unit in that case is [Ab]_eq_, referring to the fact that the observed concentration is equivalent to the Ab (often a monoclonal Ab) used for calibrating binding signals. We used known concentrations of serum Ab classes printed on microarrays as calibration curves (12, 27). Thus, while the thermodynamic concentration can be obtained without calibration, determination of the standard binding site occupancy, ${\left[Ab\right]}_{bound}^{{}^{\circ}}$, requires calibration (**Figure 11**).

The added advantage of carrying out a titration for two assay components, serum [Ab] and assay [Ag] simultaneously is that potential biases in the measurement of one can be compensated by the other. The combination of two Richards curves into a single fitting algorithm will result in a search for the intersection of the two curves at their respective inflection points, adjusting the three-dimensional surface defined by the combination of the two functions. As this is the point where both functions are changing at the fastest rate, a sensitive fitting can be obtained. Therefore, we suggest the use of dual-titration over the titration ranges expected to contain their inflection points.

## Data Availability

The raw data supporting the conclusions of this article will be made available by the authors, without undue reservation.
