# Supplementary material for: Application of the Richards function to serum antibody titration
Source: Front Immunol. 2025 Dec 12;16:1657633. doi: 10.3389/fimmu.2025.1657633 (PMC12741157; doi:10.3389/fimmu.2025.1657633)
Supplement: Supplementary file 1 [file Table1.pdf]

Supplementary

Parametrization	Inflection point	Differential equation
**Logistic**		
$L_b(x) = \dfrac{A}{1 + be^{-kx}}$	$\dfrac{1}{k}\ln(b)$	$L'(x) = kL(x)\left(1 - \dfrac{L(x)}{A}\right)$
$L_{x_i}(x) = \dfrac{A}{1 + e^{-k(x-x_i)}}$	x_i	
**Gompertz**		
$G_b(x) = Ae^{-be^{-kx}}$	$\dfrac{1}{k}\ln(b)$	$G'(x) = -kG(x)\ln\left(\dfrac{G(x)}{A}\right)$
$G_{x_i}(x) = Ae^{-e^{-k(x-x_i)}}$	x_i	
**Richards**		
$R_b(x) = \dfrac{A}{(1 + be^{-kx})^m}$	$\dfrac{1}{k}\ln(mb)$	$R'(x) =$ $mkR(x)\left(1 - \left(\dfrac{R(x)}{A}\right)^{\frac{1}{m}}\right)$
$R_{x_c}(x) = \dfrac{A}{(1 + e^{-k(x-x_c)})^m}$	$x_c + \dfrac{1}{k}\ln(m)$	
$R_d(x) = A\left(1 + (d-1)e^{-k(x-x_i)}\right)^{\frac{1}{1-d}}$	x_i	$R_d'(x) =$ $\dfrac{k}{d-1}R_d(x)\left(1 - \left(\dfrac{R_d(x)}{A}\right)^{d-1}\right)$
$R_\nu(x) = A\left(1 + \nu e^{-k(x-x_i)}\right)^{-\frac{1}{\nu}}$	x_i	$R_\nu'(x) =$ $\dfrac{k}{\nu}R_\nu(x)\left(1 - \left(\dfrac{R_\nu(x)}{A}\right)^{\nu}\right)$

Table S.1 Different parameterizations of the Richards function ($A > 0, b > 0, k > 0, m > 0, d > 1$ and $\nu > 0$) along with its special cases: the logistic function (Richards function with $m = 1$, $d = 2$ or $\nu = 1$) and the Gompertz function (Richards function when $d \to 1$ or $\nu \to 0$). For each function, the inflection point and the differential equation that the sigmoid function satisfies are given.

**Parametrization** ($f(x)$)	**Relative growth rate** $\left(\frac{f'(x)}{f(x)}\right)$	**Inflection rate** $\left(\frac{f(x_\mathrm{i})}{A}\right)$
**Logistic**		
$L_b(x) = \dfrac{A}{1+be^{-kx}}$	$k\left(1-\dfrac{L(x)}{A}\right)$	$\dfrac{1}{2}$
$L_{x_i}(x) = \dfrac{A}{1+e^{-k(x-x_i)}}$		
**Gompertz**		
$G_b(x) = Ae^{-be^{-kx}}$	$-k\ln\left(\dfrac{G(x)}{A}\right)$	$\dfrac{1}{e}$
$G_{x_i}(x) = Ae^{-e^{-k(x-x_i)}}$		
**Richards** *		
$R_b(x) = \dfrac{A}{(1+be^{-kx})^m}$	$km\left(1-\left(\dfrac{R(x)}{A}\right)^{\frac{1}{m}}\right)$	$\left(\dfrac{m}{m+1}\right)^m$
$R_{x_c}(x) = \dfrac{A}{(1+e^{-k(x-x_c)})^m}$		
$R_d(x) = A\left(1+(d-1)e^{-k(x-x_i)}\right)^{\frac{1}{1-d}}$	$\dfrac{k}{d-1}\left(1-\left(\dfrac{R_d(x)}{A}\right)^{d-1}\right)$	$d^{\frac{1}{1-d}}$
$R_\nu(x) = A\left(1+\nu e^{-k(x-x_i)}\right)^{-\frac{1}{\nu}}$	$\dfrac{k}{\nu}\left(1-\left(\dfrac{R_\nu(x)}{A}\right)^{\nu}\right)$	$(\nu+1)^{-\frac{1}{\nu}}$

Table S.2 Different parameterizations of the Richards function ($A > 0, b > 0, k > 0, m > 0, d > 1$ and $\nu > 0$) along with its special cases: the logistic function (Richards function with $m = 1$, $d = 2$ or $\nu = 1$) and the Gompertz function (Richards function when $d \to 1$ or $\nu \to 0$). For each function, the relative growth rate and inflection rate are given.

* Richards inflection rate is in $\left(\frac{1}{e}, 1\right)$

**Parametrization**	**Maximal slope** ($f'(\text{inflection\_point})$)
**Logistic**	
$L_b(x) = \frac{A}{1+be^{-kx}}$	$Ak\frac{1}{4}$
$L_{x_i}(x) = \frac{A}{1+e^{-k(x-x_i)}}$	
**Gompertz**	
$G_b(x) = Ae^{-be^{-kx}}$	$Ak\frac{1}{e}$
$G_{x_i}(x) = Ae^{-e^{-k(x-x_i)}}$	
**Richards***	
$R_b(x) = \frac{A}{(1+be^{-kx})^m}$	$Ak\left(\frac{m}{m+1}\right)^{m+1}$
$R_{x_c}(x) = \frac{A}{(1+e^{-k(x-x_c)})^m}$	
$R_d(x) = A\left(1+(d-1)e^{-k(x-x_i)}\right)^{\frac{1}{1-d}}$	$Ak\left(\frac{1}{d}\right)^{\frac{d}{d-1}}$
$R_\nu(x) = A\left(1+\nu e^{-k(x-x_i)}\right)^{-\frac{1}{\nu}}$	$Ak\left(\frac{1}{\nu+1}\right)^{\frac{\nu+1}{\nu}}$

Table S.3 Different parameterizations of the Richards function ($A > 0, b > 0, k > 0, m > 0, d > 1$ and $\nu > 0$) along with its special cases: the logistic function (Richards function with $m = 1$, $d = 2$ or $\nu = 1$) and the Gompertz function (Richards function when $d \to 1$ or $\nu \to 0$). For each function, the maximal slope is given.

* Richards maximal slope is in $(0, A)$

5-parameter models	4-parameter models
**Richards function $\left(R_\nu(x)\right)$ + constant**<br>$$const + A\left(1 + \nu e^{-k(x-x_i)}\right)^{-\frac{1}{\nu}}$$<br>parameters: $const, A, \nu, k, x_i$	**Logistic function $\left(L_{x_i}(x)\right)$ + constant**<br>$$const + \frac{A}{1 + e^{-k(x-x_i)}}$$<br>parameters: $const, A, k, x_i$
**5PL**<br>$$f(x) = d + \frac{a-d}{\left(1 + \left(\frac{x}{c}\right)^b\right)^g}$$<br>parameters: a, b, c, d, g	**4PL**<br>$$f(x) = d + \frac{a-d}{1 + \left(\frac{x}{c}\right)^b}$$<br>parameters: a, b, c, d
**5PL at e^x**<br>$$f(e^x) = d + \frac{a-d}{\left(1 + \left(\frac{e^x}{c}\right)^b\right)^g} =$$<br>$$= d + (a-d)\left(1 + \frac{1}{g}\left(g^{\frac{1}{b}}\frac{e^{x_i}}{c}\right)^b e^{-(-b)(x-x_i)}\right)^{-\frac{1}{\frac{1}{g}}}$$<br>$$= const + A\left(1 + \nu e^{-k(x-x_i)}\right)^{-\frac{1}{\nu}}$$<br>connection between parameters:<br>$const = d$<br>$A = a - d$<br>$\nu = \frac{1}{g}$<br>$k = -b$<br>$x_i = \ln c - \frac{1}{b}\ln g$	**4PL at e^x**<br>$$f(e^x) = d + \frac{a-d}{1 + \left(\frac{e^x}{c}\right)^b} =$$<br>$$= d + \frac{(a-d)}{1 + \left(\frac{e^{x_i}}{c}\right)^b e^{-(-b)(x-x_i)}}$$<br>$$= const + \frac{A}{1 + e^{-k(x-x_i)}}$$<br>connection between parameters:<br>$const = d$<br>$A = a - d$<br>$k = -b$<br>$x_i = \ln c$

Table S.4 Parameter mapping between the Richards function $R_\nu(x)$ and the 5-parameter logistic (5PL) function, and between the logistic function $L_{x_i}(x)$ and the 4-parameter logistic (4PL) function
